# Stress, Depression, and Occupational Injury among Migrant Farmworkers in Nebraska

**DOI:** 10.3390/safety2040023

**Published:** 2016-10-22

**Authors:** Athena K. Ramos, Gustavo Carlo, Kathleen Grant, Natalia Trinidad, Antonia Correa

**Affiliations:** 1 Center for Reducing Health Disparities, College of Public Health, University of Nebraska Medical Center, 984340 Nebraska Medical Center, Omaha, NE 68198-4340, USA; natalia.trinidad@unmc.edu (N.T.); acorrea@unmc.edu (A.C.); 2 Department of Human Development and Family Science, University of Missouri, Columbia, MO 65211, USA; carlog@missouri.edu; 3 College of Medicine, University of Nebraska Medical Center/Omaha VA Medical Center, Omaha, NE 68105, USA; Kathleen.Grant2@va.gov

**Keywords:** migrant farmworkers, farm injury, occupational injury, stress, depression, agricultural health

## Abstract

Agriculture is one of the most dangerous industries in the United States. Farmworkers, including migrant farmworkers, are at risk for work-related injuries. This study explores the association between stress, depression, and occupational injury among migrant farmworkers in Nebraska. Occupational injury was hypothesized to significantly increase the odds of farmworkers being stressed and depressed. Two hundred migrant farmworkers (mean age = 33.5 years, standard deviation (SD) = 12.53; 93.0% men, 92.9% of Mexican descent) were interviewed. In bivariate analyses, results indicated that stress and depression were positively associated with occupational injury. Two logistic regression models were developed. Occupational injury was a significant factor for depression, but not for stress. Participants who had been injured on the job were over seven times more likely to be depressed. These results highlight the interconnection between the work environment and mental health. More must be done to foster well-being in rural, agricultural communities. Improving occupational health and safety information and training, integrating behavioral health services into primary care settings, and strengthening the protections of the Migrant and Seasonal Agricultural Worker Protection Act may improve conditions for migrant farmworkers in the rural Midwest.

## 1. Background

Agriculture remains one of the most dangerous industries in the U.S. [1,2]. Farmworkers are at risk for occupational illnesses and injuries [3], and many are consistently exposed to occupational hazards such as unpredictable and harsh weather conditions, uncomfortable and unnatural body positions, repetitive movements, and chemical exposures [4–7]. According to the Centers for Disease Control and Prevention, approximately 167 agricultural workers suffer a “lost-work-time” injury every day [2].

Farmworkers may perceive injuries and pain to be a normal part of the farmworker lifestyle [8]; however, recent evidence suggests that injury and a worker’s mental health status are related [9]. Both stress and depression have been linked to occupational injuries in agriculture among both farmers and farmworkers [9–12].

## 2. Migrant Farmworkers

By definition, migrant farmworkers are individuals who work in agriculture on a seasonal basis and who establish a temporary home due to the mobile nature of their employment [13,14]. Migrant farmworkers support much of the crop production in the U.S. and contribute significantly to local, state, and national economic vitality [7,15]; however, these farmworkers represent an economically, socially, and legally vulnerable workforce [15,16]. The majority of migrant farmworkers are Latino immigrants from Mexico with increasing numbers of workers coming from Central America [1,17]. They are often undocumented [7,18], have low levels of formal education and English language proficiency [7,19,20], have significant cultural differences from the dominant U.S. culture [2], and may lack access to appropriate occupational health and safety information, training, and enforcement of regulations [6,17]. Numerous studies have documented high levels of occupational injury among migrant and seasonal farmworkers [3,4,6,7,12].

Understanding the underlying factors that contribute to stress, depression, and injuries among migrant farmworkers is imperative for successful prevention and intervention efforts [4]. Stress has been identified as a significant predictor of poor physical and mental health [18,21,22]. Psychosocial factors that impact the perception of stress among migrant farmworkers include poor family functioning, family separation, limited social support, marginalization, and the migrant farmworker lifestyle [23]. Stress has also been linked to increases in depressive symptoms [13]. Depression may impair a worker’s judgment, perception of risk, concentration, and may reduce concerns about personal safety [12]. In one recent study, depression was found to significantly increase the odds of occupational injury [12]. Research has demonstrated that people who report feeling stressed or depressed are more likely to engage in negative coping behaviors such as alcohol, tobacco, or other drug use [24,25], which again may increase safety risks.

## 3. Farmworkers in Nebraska

Nebraska has nearly 50,500 hired farmworkers [26] and is part of the west north central region [27]. Farmworkers in this region have a mean age of 36 years, 27% live in poverty, and 31% have less than a high school education [27].

Migrant farmworkers represent a small percentage of hired farmworkers in the state. According to the 2012 Census of Agriculture there were only 788 migrant farmworkers in Nebraska [26]; however, this number may be well below the actual number of migrant farmworkers in the state given potential issues related to legal documentation status and a mobile lifestyle. Therefore, the actual number of migrant farmworkers in Nebraska is unknown. Because many rural areas of the state are designated medically underserved areas [28], migrant farmworkers may lack access to appropriate health education, safety information, and healthcare services [29].

Previous research has found high rates of poor mental health including depressive symptoms among migrant farmworkers in the Midwest [13,20,30]. However, additional evidence on the associations between stress, depression, and occupational injury among migrant farmworkers in the Midwest is lacking.

## 4. Purpose

The primary goal of the present study was to examine the association of stress, depression, and occupational injuries among Latino migrant farmworkers in Nebraska who participated in the 2013 Nebraska Migrant Farmworker Health Study [31]. We hypothesized that occupational injury would significantly increase the odds of farmworkers being stressed and depressed.

## 5. Methods

### 5.1. Study Population

The research team partnered with the Nebraska Migrant Education Program to inform migrant farmworkers of the study. Participants were recruited through community meetings at farmworker camps in five central Nebraska counties between May and September 2013. To participate in the study, individuals had to be at least 19 years of age (the age of majority in the state of Nebraska), be of Hispanic/Latino descent, and currently work as a migrant farmworker in Nebraska. “Migrant farmworker” was defined as a person who was primarily employed in agriculture on a temporary or seasonal basis and had established a temporary abode in Nebraska.

Two hundred migrant farmworkers were interviewed in five central Nebraska counties: Adams, Clay, Hall, Holt, and York. The sample was 93.0% male, 92.9% Mexican or of Mexican descent, 59.1% had less than a high school diploma or its equivalent, and 75.8% were immigrants (see Table 1). The mean age of participants was 33.5 years old (*SD* = 12.53).

### 5.2. Procedures

The Nebraska Migrant Farmworker Health Study was guided by the Nebraska Migrant Health Task Force, which is a group of individuals that included both academic and community partners created specifically for this study to explore health issues among the migrant farmworker population. Individual members of the Task Force represented the University of Nebraska Medical Center’s College of Public Health, Creighton University’s Office of Multicultural Affairs, El Centro de Las Americas, Justice for Our Neighbors-Nebraska, Nebraska Migrant Education Program, and Legal Aid of Nebraska.

Data collection was conducted by four bilingual and bicultural members of the research team. A member of the research team explained the purpose of the study, informed potential participants of their rights as research participants both orally and in writing, answered questions, and obtained informed consent from those who were interested and met the inclusion criteria during community meetings. All study materials were available in English and Spanish, and participants had the option to read and answer the survey questions themselves or have the questions read to them by an interviewer. Interviews were conducted in as private a location as possible. Responses were anonymous and no personal identifiers were recorded. Participants were given $10 cash for their participation in the study, which was approved by the University of Nebraska Medical Center Institutional Review Board.

### 5.3. Measures

#### 5.3.1. Stress

Stress was measured by using the Migrant Farmworker Stress Inventory (MFWSI). This instrument was chosen because it is a reliable and valid measure of the stress inherent in a migrant farmworker lifestyle [21,30]. Participants reported how much stress they felt from each of the 39 items. Sample items included difficulty communicating in the English language, working long hours, difficulty finding a place to live, experiencing discrimination, or worrying about being deported. Each item was measured on a Likert-type scale from 1 ‘not at all stressful’ to 4 ‘extremely stressful’. A total score was obtained by summing the scores for all of the items, with higher scores indicating a greater degree of stress. The caseness threshold for the MFWSI was a score of 80 or higher based on standard practice for this instrument. Scores at or above this threshold indicate that a person may be at a greater risk for psychological problems [30]. The caseness threshold was used to dichotomize the variable into ‘stressed’ or ‘not stressed’ when used as the dependent variable in the logistic regression model. High internal consistency was found for the MFWSI in this sample (α = .96).

#### 5.3.2. Depression

Depression was measured by using the Center for Epidemiologic Studies Depression scale (CES-D). This scale was chosen because it is one of the most reliable and valid measures of depressive symptomology among the migrant farmworker population. The CES-D is a 20-item depression screening tool [32]. For each item (e.g., felt lonely, restless sleep, people disliked me, felt depressed, etc.), participants were asked to indicate how often they felt that way within the last week, and items were measured on a Likert-type scale from 0 ‘rarely or none of the time’ to 3 ‘most or all of the time’. Four items were reverse coded. A total score was obtained by summing the scores for all 20 items with a maximum possible score of 60. If four or more items were missing on the CES-D, it was not scored. A cutoff score of 16 or higher on the CES-D is indicative of depressive symptomatology, and higher scores indicate greater symptomatology. The cutoff point was used to dichotomize the variable into ‘depressed’ or ‘not depressed’ when used as the dependent variable in the logistic regression model. Adequate internal consistency was found for the CES-D in this sample (α = .85).

#### 5.3.3. Occupational Injury

Occupational injury was measured by a single question, “Have you ever been injured on the job?” Response options were dichotomous, no (0) and yes (1). If participants responded that they had been injured on the job, a series of questions about the nature of the injury followed including body part injured and how much productive time was lost due to the injury.

### 5.4. Covariates

#### 5.4.1. Substance Use

Both alcohol and tobacco use were assessed. A series of alcohol related questions were used including the Rapid Alcohol Problem Screen (RAPS4). RAPS4 is a four-item screening tool used to identify potential problem drinking [33]. A positive response on any of the items suggests that a person’s drinking is harmful and may have adverse effects on their well-being. Therefore, a dichotomized variable was created for risky drinking, no (0) and yes (1). Current smoking status was ascertained by asking participants, ”Do you now smoke cigarettes every day, some days, or not at all?” Participants who responded that they smoke every day or some days were classified as current smokers. Current smoking status was dichotomized, no (0) and yes (1).

#### 5.4.2. Demographic Variables

Age and hours worked per week were continuous variables. English language proficiency was measured by a single question, “How well do you speak English?” There were four original response options which were later dichotomized into not well or not at all (0) and well or very well (1). Education was measured by a single question, “What is the highest grade or year of school you completed?” There were six categorical response options which were later collapsed into three categories, less than high school education (0), high school graduate (1), and some college and/or technical training (2). Nativity was assessed by asking participants in what country they were born, either in the U.S. (0) or outside the U.S. (1). A series of additional demographic variables included gender, ethnicity, and annual income. Finally, participants were asked whether or not they had health insurance coverage as well as a regular healthcare provider.

### 5.5. Analytic Approach

SPSS version 23.0 was used to analyze the data. Basic descriptive statistics were calculated for all variables including mean and standard deviation for continuous variables and percentages for categorical variables. Appropriate data transformations were made. Bivariate and multivariate analyses were conducted to model the relationships between stress, depression, and occupational injury. First, reliability of the MFWSI and CES-D was assessed. Correlations to measure associations between the study variables and ensure non-collinearity were calculated. Continuous variables including the MFWSI, CES-D, and age were centered. Then, two multivariate logistic regression models were developed, and variables that did not have any significant bivariate associations were removed. Logistic regression model 1 was used to assess the odds of being stressed while controlling for depression, occupational injury, alcohol use, current smoking, and other demographic factors such as age, education, and English language proficiency. Logistic regression model 2 was used to assess the odds for depression while controlling for stress, occupational injury, alcohol use, current smoking, and other demographic factors such as age, education, and English language proficiency.

## 6. Results

Descriptive analyses were conducted to summarize levels of stress, depressive symptoms, and work-related injuries. Nearly one-third of participants reported a high level of stress as indicated by a score of 80 or above on the MFWSI. Depressive symptomatology was prevalent, with 45.8% of respondents having scores above 16 on the CES-D. Nearly one-fifth (18.6%) of respondents had experienced an occupational injury in their lifetime. Of those who had been injured, 34.7% had lost work time due to these injuries. The five most reported injury sites were: (1) back (38.5%), (2) foot (33.3%), (3) hand/wrist (17.8%), (4) head (17.8%), and (5) leg, knee, or hip (13.3%).

As seen in Table 2, stress was positively correlated with depression and age, *p* < .01, and it was negatively correlated with education and being at risk for problem drinking, *p* < .05. Depression was associated with stress and being at risk for problem drinking, *p* < .01. Occupational injury was positively correlated with both stress and depression, *p* < .01, as well as age, *p* < .05.

Multivariate logistic regression analyses were conducted to further examine the relationships between stress, depression, and occupational injury (see Table 3). All independent variables were entered simultaneously in each model. Both models were good fits for the data as indicated by non-significant Hosmer and Lemeshow tests.

### Model 1, Stress

Depression, *p* = .002, and education, *p* = .02, were significant factors for stress. Age was marginally significant, *p* = .07. For each unit increase in depression, participants were 2.5 times more likely to also be stressed. Occupational injury was not a significant factor for stress in this sample.

### Model 2, Depression

Stress, *p* = .01, occupational injury, *p* = .02, and education, *p* = .04, were significant factors for depression. Being at risk for problem drinking was marginally significant, *p* = .06. Participants who had experienced an occupational injury were 7.35 times more likely to be depressed than those who had not experienced an occupational injury.

## 7. Discussion

In bivariate analyses, occupational injury was positively associated with both stress and depression. The hypothesis that occupational injury significantly increases the odds of being depressed, over and above the effects of several possible confounds including smoking, drinking, nativity, education, and age was supported. However, occupational injury was not found to be a significant factor for stress. The present findings are consistent with the Mexican Immigration to California: Agricultural Safety and Acculturation (MICASA) study that found a significant association between depression and injury in a sample of Mexican farmworkers in California [12]. Other studies of farmers and farmworkers have found this significant effect as well [9]. Taken together, the prior and present findings suggest that reducing occupational injuries can reduce depressive symptoms among Latino migrant farmworkers.

Of further interest were the prevalence rates of stress, depressive symptoms, and injuries in this sample. Results demonstrated that nearly one out of every three workers reported relatively high levels of stress, nearly one out of every two workers reported relatively high depressive symptoms (compared to only 7.6% of the general population [34]), and approximately 19% reported injuries (compared to 3.3 injuries per 100 workers in the general population [35]. These prevalence rates suggest a psychologically and physically risky work environment. From a business perspective, the 34.7% of injuries that resulted in lost work time present a challenge to this industry. It is important to ensure that job-related training is provided and proper safety equipment is available. Furthermore, understanding the risk and protective factors associated with psychological and physical well-being, health, and safety are of great importance. Moreover, identification of such factors presents opportunities for intervention efforts to improve health and safety outcomes among migrant farmworkers, as well as, productivity outcomes within the agricultural industry.

Because a majority of the workers in this sample noted that they did not have a primary care provider, it is necessary to find ways to use the few resources that rural communities and migrant farmworkers can access to promote safety and well-being. For example, research has shown that social support can mitigate depressive symptoms [36]. Interventions to increase social support and foster integration among workers themselves or between workers and communities could be beneficial. Additionally, because Latinos value family and communal ties [37], facilitating the development and maintenance of strong transnational ties with family and friends in the worker’s country of origin may also be beneficial.

Farmworkers have limited access to healthcare services [29]. However, recognizing, screening, and treating mental health issues among migrant farmworkers is imperative [10]. Because Latino farmworkers often limit their usage of these services to only when in extreme need, integration of behavioral health services into primary care settings is important. Latinos often prefer to use primary care providers to address mental health concerns and integration could then streamline the process for accessing appropriate services [12]. Primary healthcare providers in migrant health centers, rural clinics, and hospitals should conduct mental health assessments using standard screening tools such as the CES-D as a standard of practice, especially in regards to follow-up after an occupational injury. If concerns are found, then providers should make appropriate referrals to existing behavioral healthcare services; however, if local services are not available, a referral to a hotline such as the Nebraska Rural Response Hotline, could be made.

Unfortunately, rural communities are disproportionately underserved [28,37]. Strengthening the healthcare professional pipeline and ensuring that rural communities have access to behavioral health professionals is important. Increasing the number of bicultural and bilingual healthcare providers is essential given the growth of the Latino population in new destination areas of the Midwest [38]. These providers may be more aware of the stigma associated with mental health disorders among the Latino migrant farmworker population and understand the types of interventions that may be needed to address mental health concerns in a culturally and linguistically appropriate manner.

Furthermore, implementing health and safety education and outreach initiatives could also aid in identifying workers suffering from job-related injuries and mental health concerns as well as connect them with appropriate resources. Winkelman, Chaney, and Bethel (2013) indicate that migrant health centers that provide mental health counseling should also provide supportive services such as assistance with documentation issues, transportation, and provision of English courses for workers to reduce stress and depressive symptoms [25]. Collaborations could be developed to increase access to these supportive services such as a medical-legal partnership focused on immigration-related legal issues. Medical-legal partnerships can improve health by addressing legal concerns that impact a person’s ability to access and pay for healthcare [39], such as by helping individuals apply for legal status through means like the T-visa (for victims of human trafficking including labor trafficking) [40]. Regardless, it is clear that communities need to explore opportunities to improve access to social, physical, and mental health services and institute strategies that reduce barriers to culturally and linguistically appropriate occupational health and safety information and training.

These findings also have important implications for policy as well. The Migrant and Seasonal Agricultural Worker Protection Act (MSPA) needs to be strengthened and enforced to ensure that workers are provided information in their primary language about the job contract, payment, and workers’ compensation. Because Nebraska’s thriving agricultural industry may attract a significant number of immigrant and migrant farmworkers, advocates, healthcare providers, communities, and workers must be vigilant about protecting human, civil, and labor rights. Although Nebraska does not provide workers’ compensation to every farmworker [41], this protection should be extended, especially to migrant and seasonal farmworkers. Currently according to Nebraska law, only farms with 10 or more full-time employees working for at least 13 weeks per year must provide this coverage [41,42]. If an exempt employer chooses not to provide workers’ compensation coverage, written notification must be provided to employees at the time of hire informing them that they are not covered by the NebraskaWorkers’ Compensation Act and will not be compensated under the act if they are injured on the job [42]. Without workers’ compensation coverage, farmworkers may return to work sooner than they should after an injury and their condition sometimes worsens, which may in turn lead to depression. Healthcare providers have a duty to understand the workers’ compensation system in their state and assist with claims if injured farmworkers have access to this benefit. Stronger policies and enforcement can promote well-being, reduce occupational injuries, and improve overall safety among this vulnerable population.

### Limitations

This study was cross-sectional in nature; therefore, causation cannot be determined. Experiencing an injury could have led to depression, but depression could have also led to injury. Longitudinal research is needed to clarify the causal pathway. This study had several other limitations including the reliance on self-reported data, which may be prone to self-presentational demands and shared method bias. Moreover, given the physical location of the data collection process, it was not always possible to ensure confidentiality, and data was collected with both workers and crew chiefs (supervisors) present, which may have impacted participant responses. Because of the study design, these results may not be representative of all migrant farmworkers in Nebraska. Future research is needed to obtain data from multiple sources (e.g., medical records, workers’ compensation data, and insurance claims) and in private, confidential settings to reduce such biases. In addition, many farmworkers still do not know their rights, and there is still a problem with incomplete reporting of occupational injuries and illnesses [43]. Workers may not understand the concept of “work-related injury” because there is not a standard translation in Spanish [4], and therefore may be less likely to report an injury. There are also significant issues related to documentation status, fear of retaliation, lost wages, and cultural issues such as “machismo” that may impact reporting. Finally, injury data may be underreported due to recall bias.

## 8. Conclusions

Occupational injuries affect, not only physical health, but also mental health including stress and depression. This study explored the association between stress, depression, and work-related injuries. It is clear that more needs to be done to improve the health, safety, and well-being of migrant farmworkers in Nebraska. Local, state, and federal policy reforms including comprehensive immigration reform, may be necessary to create the conditions for all farmworkers to thrive. Future research may explore the role of acculturation in mediating stress and depression as well as the relationship between occupational injury and depression among both crop production workers and livestock production workers in the Midwest.

## Figures and Tables

**Table 1:** Demographic Characteristics of Respondents.

Variable	N (%)
Gender	
Male	185 (93.0)
Female	14 (7.0)

Age	
Under 25	62 (33.7)
25–40	66 (35.9)
Over 41	56 (30.4)

Education	
Less than High School Diploma or Equivalent	114 (59.1)
High School Graduate or Higher	79 (40.9)

Ethnicity	
Mexican	184 (92.9)
Other Latino	14 (7.1)

Nativity	
Born in the United States	45 (24.2)
Born outside the United States	141 (75.8)

Annual Income	
Less than $10,000	110 (61.5)
$10,000–$15,000	32 (17.9)
More than $15,000	37 (20.6)

Hours Worked Per Week	
35 or Less	33 (18.5)
35–50	110 (61.8)
More than 50	35 (19.7)

Have Health Insurance Coverage	
Yes	35 (18.6)
No	135 (71.8)
Don’t Know	18 (9.6)

Have a Primary Care Provider	
Yes	20 (10.6)
No	157 (83.1)
Don’t Know	12 (6.3)

**Table 2:** Correlations among Study Variables

	Occupational Injury	Stress	Depressed	Age	Nativity	English Proficiency	Education	Type of Farmwork	Hours Worked/Week	Health Insurance	At Risk for Problem Drinking	Current Smoker
Occupational Injury	1.00											
Stress (*M* = 62.32, *SD* = 36.41)	0.23**	1.00										
Depressed (*M* = 17.05, *SD* = 10.36)	0.20**	0.49**	1.00									
Age (*M* = 33.51, *SD* = 12.53)	0.16*	0.30**	0.10	1.00								
Nativity	−0.02	0.03	−0.10	0.23**	1.00							
English Proficiency	0.04	0.06	0.09	−0.11	−0.61**	1.00						
Education	−0.07	−0.17*	−0.06	−0.31**	−0.04	0.13	1.00					
Type of Farmwork	−0.04	0.02	−0.02	0.03	−0.09	0.11	−0.05	1.00				
Hours Worked/Week	0.07	0.12	0.03	0.05	0.00	0.11	0.07	0.08	1.00			
Health Insurance	0.06	−0.14	−0.02	−0.13	0.02	0.03	0.02	0.11	−0.11	1.00		
At Risk for Problem Drinking	−0.11	−0.20 *	−0.26 **	−0.09	0.03	0.09	0.06	0.18	0.06	−0.10	1.00	
Current Smoker	0.03	−0.04	0.08	−0.11	−0.04	0.04	0.10	−0.04	0.15	−0.02	−0.26**	1.00

* *p* < .05;

** *p* < .01.

**Table 3:** Adjusted Odds Ratios and 95% Confidence Intervals (CIs) for Relationships Between Stress, Depression, and Occupational Injury Among Migrant Farmworkers in Nebraska.

	Model 1: Stress				Model 2: Depression			

	B	S.E.	Exp (B)	95% CI	B	S.E.	Exp(B)	95% CI
Stress					0.03	0.01	1.03 **	1.01, 1.05
Depression	0.92	0.30	2.50 **	1.38, 4.52				
Occupational Injury	0.32	0.79	1.38	0.30, 6.41	2.00	0.86	7.35 *	1.35, 39.93
Age	0.05	0.03	1.05 +	1.00, 1.10	0.01	0.02	1.01	0.96, 1.06
English Proficiency	0.68	0.69	1.97	0.51, 7.66	0.57	0.57	1.78	0.58, 5.47
Education	−1.16	0.48	0.31 *	0.12, 0.80	0.72	0.35	2.06 *	1.04, 4.06
At Risk for Problem Drinking	−0.23	0.67	0.80	0.22, 2.93	−1.00	0.53	0.37 +	0.13, 1.04
Current Smoker	*−*0.50	0.66	0.61	0.17, 2.22	0.61	0.52	1.83	0.66, 5.09
Constant	*−*3.20	1.63	0.41		0.31	0.91	1.37	

+ *p* < .10;

* *p* < .05;

** *p* < .01.
